# A multimodal discourse analysis of select Indian queer advertisements and representation of negotiated queer visibility in mass media

**DOI:** 10.3389/fsoc.2026.1857542

**Published:** 2026-07-17

**Authors:** S. S. Aswani, R. Preetha

**Affiliations:** Department of English, School of Social Sciences and Languages, VIT Chennai, Chennai, India

**Keywords:** commodification, decriminalization, LGBTQ+ visibility, MCDA, queer advertisements, rainbow capitalism, Section 377

## Abstract

**Introduction:**

The queer portrayals in mainstream advertisements have significantly increased after India scrapped Section 377 on 6th September 2018. But only a few scholarly studies have been made on how these marketing campaigns have positively portrayed queer identities by uplifting and embracing diversity, rather than merely being profit-oriented.

**Methods:**

This study focuses on analyzing advertisements in India featuring LGBTQ+ characters post 2018, using a mixed-method design. Applying the Multimodal Critical Discourse Analysis, five advertisements are studied, and along with this, a survey of 100 participants is included on the visibility, normalization, and commodification.

**Results:**

The survey results suggest that queer advertisements are socially constructive by heightening the understanding and destigmatizing LGBTQ+ identities despite being driven by commercial reasons. The multimodal discourse analysis highlights that these queer advertisements often depict the affirmative narratives of common residential settings, familial acceptance, and emotional interaction, basically pushing queer inclusion.

**Discussion:**

An ambivalent yet culturally significant shift is found as queer advertisements function as a form of negotiated visibility in which cultural inclusion and commercial intent coexist. It feels as though the companies are trying to address a social issue while making a buck for themselves. The paper contributes to the ongoing debates on queer visibility and highlights the transformative potential and representational limits in Indian Queer advertisements after 2018.

## 1 Introduction

The longstanding presence of gender and sexual diversity within Indian subcontinent is reflected in a range of historical records, mythological narratives and cultural artifacts. Several narratives within Hindu mythology have been interpreted as expressions of gender fluidity and non-binary identities. Lord Shiva, depicted in the form of Ardhanarishvara, a composite deity embodying both masculine and feminine principles remains as an interpretation of queer identities in modern times. There are several other characters, such as Shikandi and Arjuna from the Mahabharata, whose genders are altered. Lord Vishnu also took the Mohini avatar and had a child, Ayyappan, with lord Shiva. The temple carvings in India, such as those of the Khajuraho of Madhya Pradesh, built during the 10th century, have been interpreted by contemporary scholars and activists as indications of historical recognition of gender and sexual diversity (Painted with Pride, 2024). Historical accounts of Babur's admiration for a young man named Baburi have also been cited by scholars as evidence of the presence of same-sex desire within South Asian historical records.

These examples although demonstrate the historical presence and cultural visibility of gender and sexual non-conformity in the Indian subcontinent should not be interpreted as indicative of universal or unconditional social acceptance. A deeper examination of socio-legal and cultural context is necessary for a better understanding. In *Same-Sex live in India: Readings from Literature and History*, Vanita and Kidwai (2000) present a critical analysis, documenting that precolonial India. Despite the absence of the widespread and institutionalized homophobic rhetoric which was eventually formalized under British rule, precolonial India did not offer an environment of unfettered acceptance. Their research reveals that various legal and religious treatises of the era contained provisions for prescribed punishments concerning certain homosexual behaviors.

Upadhyay (2020) gives caution against the propagation of a politically convenient narrative that leads among the civilization which portrays a uniformly accepting precolonial past. Mainstream Hindu narratives which function as universal frameworks limits our ability to fully understand the lived experience of queer, trans, and gender nonconforming people, in particular the ones who were double marginalized on the basis of caste, religion, ethnicity, and class (Upadhyay, 2020). This “saffron-washing” can serve Hindu Nationalist purposes while erasing the role of Brahminical endogamic structures. The imposition of colonial rule in India and the emergence of Victorian morality through the promulgation of the Indian Penal Code (IPC) Section 377 worsened the situation by legally classifying queerness as unnatural and criminal. Thus, the British legal framework did not flourish in an entirely blank social landscape within India but rather overlaid and transformed existing societal mechanisms in to a much drastic situation.

The law that targeted homosexuals was struck down by the Delhi High Court in 2018, and there was a visible shift toward legislative inclusion. (Yadav, 2021) The rejection of the Supreme Court in 2023 to legally accept marriage equality rights is merely a milestone to obtain. Legal advancements were made in the daily lives of queer individuals, but limitations in the social, economic, and health disparities continue to exist. The health researchers say that there are several inhumane repercussions which treat the homosexuals as downtrodden species. They include harsh acts such as restrictions on basic medical needs, exclusion by insurance, higher mental burdens, and a lack of parental and partnership relationship status for queer people (Rosa et al., 2024). Thus, the representation of queer identities in mainstream commercial advertising has experienced a landmark transformation in India. Several brands within India have positioned LGBTQ+ narratives in the national discourse and infused emotional appeals to reach the majority. Despite the progressive surface, as Walters (2021) argues these inclusive advertisements may have an underlying tension surrounding commodification which may eventually warrant questioning. It is within this context that the present study examines queer representation in Indian commercial advertising. The analyzation of these commercials will further investigate whether such representations contribute to meaningful social inclusion. or primarily function as market-driven strategies

## 2 Literature review

### 2.1 MCDA in contemporary research

Multimodal Critical Discourse Analysis (MCDA) is an emerging approach in qualitative data analysis, which expands Critical Discourse Analysis (CDA). It also includes visual and auditory elements rather than being rooted just in the linguistic aspect, which interact with each other to provide a deeper meaning, challenging the power relations in the contemporary context (Machin, 2013). CDA focuses only on the language in texts and speeches. Being a part of the multimedia world, it is necessary to understand that meaning is derived from various elements collaborating. This shift in perspective is called the “multimodal turn.” It is important to analyze more than just words while studying how people communicate and make sense of things. MCDA is used to assess global policy narratives critically.

### 2.2 Theoretical foundation

Systematic Functional Linguistics (SFL) provides the groundwork for Multimodal Critical Discourse Analysis (MCDA) which is incorporated to the primary analysis of the study. A pioneer in SFL named Michael Halliday has significantly contributed to the evolution of linguistic thought. His contribution to SFL systematically expands and reinterprets the concepts proposed by his mentor, J. R. Firth, back in the 1960s. Kress and Theo van Leeuwen's social semiotic works such as *Reading Images* (1996) and *Multimodal Discourse* (2001) represent an important benchmark in discourse studies. Their works enumerates that the traditional understanding of language which acts as the primary source of meaning moves beyond its reach. They state that visual communication is much efficiently being used in ways governed by rules and conventions different from those of language, thus necessitating a grammar of visual design that operates independently of linguistics structures. This leads to the understanding that language alone cannot account for the full complexity of meaning-making processes, as communication is also mediated through visual, auditory, and spatial resources. MCDA develops a methodological framework aimed at denaturalizing the communication process in response to this limitation.

### 2.3 Application of MCDA

The multimodal approach to discourse emphasizes that language cannot be found in isolation, as it is always embedded within a broader semiotic environment. In the YouTube video, which includes the selected advertisements, the language is accompanied by a sequence of scenes. As mentioned before, meaning-making emerges from the interaction of multiple modes that include visual, spatial and material elements rather than being rooted solely in linguistic structure. MCDA has been applied across diverse research contexts around the world. Starting by analyzing university management documents (Ledin and Machin, 2020) to examining media coverage of social injustice Machin and Mayr (2025). Social injustice, especially among the homosexuals and the marginalized in news coverage can also be brought to light using the methodology of MCDA in the field of Media Studies. The discussion of the Roma campus eviction in Machin and Mayr (2025) study serves as a significant example of critical discourse analysis, demonstrating how institutional language can obscure the realities of an event. Their analysis reveals the discrepancy between the neutral and bureaucratic framing of the eviction and the actual experiences of those affected, highlighting how linguistic choices shape public perception and can conceal underlying power relations.

### 2.4 Representation of LGBTQ+ identities

The content analysis of Indian advertisements conducted by Jetubhai (2024) proves that there is a notable surge in queer representation post-2018. The finding of this study marked a disparity of homosexual cisgendered characters often depicted in urban, affluent settings framed by “Theme of Love,” while transgender characters are often seen fighting for human rights in non-urban environments. The study on Arts-Based Health Research integrated MCDA with “cellphilming” to examine how GBTQ men who are into pup play feel about their bodies. The interpretation of the participant's semiotic choices reveals how subcultural spaces provide a “therapeutic escape” from cis-heteronormative body standards (Joy et al., 2024).

### 2.5 Rainbow capitalism and corporate pride activism

The commercialization of social movements, especially LGBTQ+ activism, has been severely criticized as “rainbow capitalism,” where companies take on progressive symbols but maintain exploitative practices (Walters, 2021). Brands frequently engage in “Pinkwashing” during pride month. They use rainbow branding to signal their support without providing meaningful workplace protection or financial support for queer communities (Sender, 2004). Although LGBTQ+ symbols and narratives are incorporated for marketing during pride month, priority often falls on brand equity than political advocacy (Duggan, 2002). Sender (2004) traces that commodification has historically shifted from avoiding queer consumers to actively courting them, a globalized phenomenon. Decriminalization of same-sex relations allowed brands a low-risk ally posture. The post-decriminalization surge in Queer advertisements mirror what Gould (2009) identifies as “strategic visibility” leverages marginalized identities for reputational capital during specific periods, without year-round commitment to structural change. Duggan's (2002) concept of homonormativity is central to understanding this phenomenon as corporate Pride campaigns tend to favor narratives of romantic love, family acceptance, and individual self-expression that are palatable to mainstream heterosexual audiences, while sidelining demands for legal equality.

The inclusion of queer imagery can be performative, projecting an appearance of openness while failing to address fundamental issues of exclusion (Ahmed, 2012). This study views Indian corporate Pride advertising as a negotiation site between visibility and commodification.

### 2.6 Existing gap in MCDA

The identified gaps in Literature review include methodological fragmentation and the under-representation of certain semiotic modes. As Ledin and Machin argue that MCDA has remained fragmented since it's emergence in the 1990s, they emphasize on the necessity of integrating existing theoretical and empirical work with visual, auditory, and material aspects of communication to foster a more comprehensive understanding of multimodal interaction. Research concerning queer representations within Indian advertisements remains under-explored. Indian media studies reveal a significant lack of representation of rural LGBTQ+ communities (Jetubhai, 2024).

## 3 Method details

This article uses a sequential mixed-methods design for analysis. A survey is collected for quantitative analysis which quantifies public attitude toward queer portrayal in media, corporate support, and the perceived legitimacy of commercialized queer representations. The MCDA of five selected advertisements is conducted next focused on examination of the specific semiotic strategies through which rainbow capitalism operates in practice. Both the observations of quantitative and qualitative analysis are compared to draw inferences on queer portrayals in Indian advertising and visibility as empowering or exploitative from an Indian context.

### 3.1 Survey design and administration

A nine-item Likert scale questionnaire (ranging from Strongly Agree to Strongly Disagree) was developed to quantify public attitudes toward queer portrayal in media, corporate Pride activism, and the perceived legitimacy of commercialized queer representations. The questionnaire was administered digitally via Instagram and WhatsApp using convenience sampling, yielding 100 responses from participants aged 17–40 across varied educational backgrounds. Though the convenience sampling limits the generalizability of findings, it was deemed appropriate given the study's scope. Similarly, the demographic profile of respondents, predominantly young media consumers find relevance to the study's focus on contemporary queer media visibility.

### 3.2 Multimodal critical discourse analysis

The analytical process involves close and repeated engagement with each advertisement as a complete multimodal text. The researcher pays close attention to the interaction between linguistic, visual, and auditory modes rather than treating each in isolation. This integrative reading practice is central to MCDA methodology, as meaning in multimodal texts emerges from the simultaneous interaction of multiple semiotic resources rather than from any single mode alone (Kress and van Leeuwen, 2011). Machin and Mayr (2025) caution against mechanically applying a predetermined checklist of features, which emphasizes that analytical categories must remain responsive to the specific semiotic environment of each text. Kress and van Leeuwen's (2011) grammar of visual design provides consistent analytical anchors across all five advertisements. It also encompasses representational, interactive, and compositional meanings that structure the visual analysis.

### 3.3 Addressing interpretative bias

The trustworthiness of this study's qualitative findings is grounded in Lincoln and Guba's (1985) methodological triangulation which is identified as a primary means of establishing credibility in qualitative inquiry. The convergence of MCDA findings with quantitative survey data allows each strand to corroborate and interrogate the other. This produces a much more robust analytical account than either methods could achieve alone. Dependability is ensured through the consistent application of Machin and Mayr's (2025) three-stage analytical framework across all five advertisements ensures dependability, making the analytical process transparent and replicable in principle. The positionality of authors as researchers situated within the Indian sociocultural context enriches cultural interpretation.

## 4 Quantitative approach

### 4.1 Respondents' profile

The demographic characteristics of the participants are demonstrated in Table 1. The sample consists of 100 respondents drawn from varied age groups and educational backgrounds, recruited through convenience sampling via Instagram and WhatsApp. The gender distribution among the participants includes 61% of women and 39% of men. Notably, no participants identified as third gender, which constitutes a limitation of the sample given the study's focus on queer representation. Although age-specific statistical analysis is not explicitly incorporated in the study, the respondents broadly represent the age group of 17–40, which is typically more engaged with contemporary digital and media culture. The sample spans educational backgrounds from secondary schooling to doctoral level, as detailed in Table 1.

**Table 1:** Demographic characteristics of respondents.

Measure	Item	Frequency	Percentage
Gender	Female	61	61%
	Male	39	39%
Age	17–19	28	28%
	20–25	61	61%
	26–30	10	10%
	Above 30	1	1%
Qualification	Doctoral degree	10	10%
	Master's degree	40	40%
	Professional degree	9	9%
	Bachelor's degree	15	15%
	School education	25	25%
	Others	1	1%

The questionnaire was circulated through Instagram and WhatsApp using convenience sampling, targeting digitally engaged media consumers relevant to the study's focus. As it limits the extent to which the results can be generalized it also allows the collection of diverse responses across different demographic groups. The existing demographic composition is directly relevant to the study, as it focuses on the perception of queer representation and commodification in the post-decriminalization Indian media landscape.

### 4.2 Descriptive statistics

The quantitative analysis is conducted using IBM SPSS Statistics. The responses collected were subjected to descriptive statistical analysis to map the general response pattern of the audience to queer-themed media. The demographic characteristics of the respondents is examined using frequency analysis. To assess the internal consistency of the scale, Cronbach Alpha reliability test is conducted. Exploratory Factor Analysis (EFA) using Principal Component Analysis with Varimax rotation is performed for constructing validity. To examine the relationship between variables Pearson Correlation Analysis is done, identifying the strength and direction of association among different questionnaire items. This research article integrates quantitative and qualitative findings to draw inference on the understanding of neoliberal ideology in queer media representation within everyday discourse.

### 4.3 Reliability and validity tests

The reliability of the study's survey instrument was evaluated using Cronbach Alpha test in SPSS. The yielded Cronbach Alpha coefficient is 0.780 for the nine-item scale is illustrated on Table 2. A coefficient of 0.70 is generally considered satisfactory in social science research. Thus, the obtained Cronbach Alpha value is acceptable, indicating the questions are reliable to measure the respondent's perception of queer visibility and corporate involvement. The stability of the scale is demonstrated by the Item-Total Statistics as no substantial improvement or decline in the alpha value is noted upon item deletion. This suggests that no single item significantly undermines the reliability of the scale.

**Table 2:** Reliability and validity analysis.

Item	Cronbach's alpha	Factor loading		*p*-Value
		Factor 1	Factor 2	
Media consumption	0.767	–	0.704	0.000
Representation improved	0.788	–	0.757	0.000
Corporate pride genuine	0.755	–	0.522	0.000
Pride products positive	0.736	–	0.725	0.000
Straight audience appeal	0.750	0.607	–	0.000
Limited identities focus	0.750	0.573	–	0.000
Commercialization dilutes meaning	0.736	0.534	0.558	0.000
Profit-driven branding	0.769	0.845	–	0.000
Pride marketing exploitative	0.770	0.765	–	0.000

Cronbach's α = 0.780; KMO = 0.707; Bartlett's Test *p* < 0.001.

The validity of the survey instrument was primarily assessed through Exploratory Factor Analysis (EFA) using Principal Component Analysis (PCA). It is a common method for establishing construct validity by identifying the underlying structures within the data. The suitability of the dataset for factor analysis is tested using Kaiser–Meyer–Olkin (KMO) value. The obtained result of KMO test is of 0.707. A KMO value above 0.6 is considered acceptable in statistical practice, indicating the sample size and correlations between items are adequate for factor analysis. Bartlett's Test of Sphericity is highly significant (χ^2^ = 277.646, *p* < 0.001), confirming that the variables in the study are sufficiently correlated to be grouped into factors. Components with eigenvalues >1 are usually considered important and components 1 and 2 clearly meet the criteria as illustrated in Table 3.

**Table 3:** Total variance explained.

Component	Initial eigenvalue (total)	Initial % of variance	Initial cumulative %	Rotated % of variance	Rotated cumulative %
1	3.384	37.603	37.603	28.757	28.757
2	1.649	18.321	55.924	27.167	55.924

The PCA with Varimax rotation extracted two distinct components that together explain 55.92% of the total variance. This structural split demonstrated that the survey successfully differentiates between different dimensions of the topic as illustrated in Table 2. The first factor captures critical perspective on commercialization defined by items regarding profit-driven motives (0.845) and exploitative marketing (0.765) indicating a strong and distinct construct related to skepticism toward corporate involvement. The second factor comprises items related to positive perception of queer representation including media consumption (0.704), perceived improvement in representation (0.757) and acceptance of corporate initiative such as Pride-themed products (0.725), suggesting a coherent construct centered on normalization and acceptance. The result of the factor analysis confirms the construct validity of the research as the items are clustered clearly into two logical groups. It demonstrates that the questionnaire was accurately measuring these two distinct perceptions rather than a single confused sentiment. The individual communalities are all initially set to 1.000, confirming that each of the nine items contribute significantly to the overall model.

The correlation analysis demonstrated in Table 4 reveals a complex interplay between acceptance and critique. It indicates that the increased engagement with queer media is associated with both positive perception of representation and heightened skepticism toward its commercialization. The correlation results show a mix of both positive and negative relationships, but the overall pattern is predominantly positive correlations.

**Table 4:** Correlation.

Construct	1	2	3	4	5	6	7	8	9
Media consumption	1								
Representation improved	0.413^**^	1							
Corporate pride genuine	0.141	0.262^**^	1						
Pride products positive	0.351^**^	0.306^**^	0.543^**^	1					
Straight audience appeal	0.259^**^	−0.010	0.373^**^	0.421^**^	1				
Limited identities Focus	0.260^**^	0.135	0.199^*^	0.419^**^	0.413^**^	1			
Commercialization dilutes meaning	0.446^**^	0.225^*^	0.335^**^	0.458^**^	0.411^**^	0.435^**^	1		
Profit-driven branding	0.091	−0.146	0.253^*^	0.130	0.382^**^	468^**^	0.305^**^	1	
Pride marketing exploitative	0.157	−0.132	0.238^*^	0.176	270^**^	0.239^*^	0.466^**^	1 0.563^**^	1

^*^p < 0.05. ^**^p < 0.01.

## 5 MCDA of selected advertisements

Commercialization is a double-edged sword, playing a huge role in shaping cultural, social, and individual identities. For the study, five advertisements (Table 5) were chosen based on a set of purposive criteria designed to ensure analytical representativeness and relevance to the post-decriminalization context.

**Table 5:** List of selected advertisements.

S. No	Brand/company	Year	Hashtags
**1**	**Borosil**	**2019**	**#FirstValentine**
**2**	**OkCupid**	**2019**	**#LoveAtFirstPride**
**3**	**Closeup**	**2020**	**#FreeToLove**
**4**	**Infosys**	**2022**	**Embrace with Love**
**5**	**Amazon Prime**	**2023**	**Happy Pride Month**

^#^Brand/company names and taglines are bolded.

All the selected advertisements were released between 2019 and 2023, immediately following the decriminalization of homosexuality through the reading down of Section 377 in 2018, ensuring reflection of the emerging phase of queer visibility in Indian commercial media. All five advertisements are publicly available on YouTube and have earned considerable viewership, ensuring their reach within mainstream media discourse rather than being niche or experimental campaigns. Diverse range of industry sectors such as FMCG and lifestyle products (Borosil and You, 2019; Closeup India, 2020), technology and corporate branding (Infosys), digital dating platforms (OkCupid India, 2019) and OTT media (Amazon Prime) which allows cross-sectoral analysis of ho rainbow capitalism operates across different brand contexts. Each advertisement explicitly features LGBTQ+ characters or narratives as central to the campaign's message, rather thanas incidental or background representation. Regional language advertisements were excluded as they constitute a distinct advertising register requiring separate cultural and linguistic contextualization. It is acknowledged that this selection does not represent rural media contexts, which remain a limitation and an avenue for future research.

### 5.1 Borosil - #FirstValentine - 2019

#### 5.1.1 Identifying semiotic resources

The advertisement follows a first-person narrative of a lesbian woman. The speaker uses negative evaluation for stereotypical romance patterns, such as “stupid romantic things” and “I didn't like any of it,” to show the past exclusion she has faced. She makes a shift in the middle, “This year it's going to be different,” proclaiming a change that has happened over time. Released shortly after the decriminalization of homosexuality, the advertisement constructs a narrative of newly legitimized public visibility for same-sex relationships. The past she had to hide is no longer a crime, and she is finally out and proud. The language used in the ad is a mixture of Hindi and English, which together state that the setting should be urban, the primary demographic targeted by most advertisements (Jetubhai, 2024). The advertisement explicitly shows a list of semiotic objects, such as roses, balloons, gifts, and a special candlelight dinner, that signify the traditional Valentine's Day setting that was denied to the speaker in the past. The interior decoration and appliances in the house portray the elegant urban environment with a lot of glassware (Jetubhai, 2024). Urban settings are highly focused on as they are the potential buyers of elegant glassware. The high-end dinner functions as a marker of middle-class urban respectability, positioning queer acceptance within a framework of consumer culture.

#### 5.1.2 Signified associations

The connotations and symbolisms behind the choices made are discussed in this part. A contrast of “hiding love” with the “FirstValentine” is used as a metaphor for the legal rights that the queer community gained after scrapping Section 377 from the Indian Penal Code. The juxtaposition of time from exclusions in the past, replaced by the “first” act of public celebration, portrays the social visibility. The ad also recontextualizes the heteronormative norms into queer narrative. It proves the legitimization of same-sex relationships and puts them in the same context of “romance” as that of heterosexual relationships.

### 5.2 OkCupid #LoveAtFirstPride - 2019

#### 5.2.1 Identifying semiotic resources

This campaign portrays India's progress from a private past to a legal and open future. The carefully chosen linguistic element includes the repetitive use of so many “first…” throughout the advertisement and the hashtag #LoveAtFirstPride. This hashtag functions as a metaphor for the social rebirth of the queer community. This advertisement presents the post-decriminalization era where once off-limit subjects are now embraced. The taglines #MatchOnWhatMatters and the slogan “Love is Love” act as a tool which moves the discourse from legal acceptance toward a universal theme of love. The questions like “What's your thought on PDA?” being asked to a gay man in the public platform by OkCupid denaturalizes the expectation of LGBTQ+ invisibility, asserting that times have finally changed. The auditory discourse provides emotional coherence making the campaign authentic. The queer character's answers being loud and proud in a confident tone is associated with gaining legal recognition. The lack of scripted plot twists in the narrative and featuring real individuals with real experience signifies the shift toward biographical legitimation. This advertisement uses confessional style which emotionally aligns with the viewers promoting a sense of familiarity. Diverse influencers like Pratul Narang and Lauren Robinson are the subjects of the advertisement. Their direct gaze into the camera talks a lot, demanding the viewers to see them as equals rather than marginalized others. The visual discourse communicates ideologies regarding who is visible in new India. The advertisements are consistently set in urban setting, reinforcing that social visibility is a privilege only to those who are financially stable and have metropolitan identity.

#### 5.2.2 Signified associations

OkCupid's campaign provides digital visibility to queer community which contrasts the previous lack of public platforms. This subtly implies that the dating app presents itself as a safe space for those who were invisible in the past. The metaphor of various “firsts” is associated with the social rebirth of the marginalized community. OkCupid uses real experiences of the influencers to bring in authenticity. The direct gaze of these influencers into the camera constitutes what (Kress and van Leeuwen 1996) term as “demand image,” a visual address that calls on viewers to acknowledge the subject as an equal, moving queer subjects from invisibility to active engagement. The profile option of gender identities acts as the semiotic resource that recontextualizes the act of matching into a designed and autonomous choice. This signifies the normalization of queer identity in mainstream society. This advertisement invalidates the pressure set by the society to hide queer identities compared to previous times.

### 5.3 Closeup #FreeToLove - 2020

#### 5.3.1 Identifying semiotic resources

The advertisement is about three couples, their journeys of love and different barriers they face. This study focuses only on the queer couple Arnav and Ashraf. “I'm Ashraf… this is my to be husband Arnav” is the opening statement of the couple demonstrating the first-person confessional narrative employed in the advertisement. This dialogues in the advertisement highlights binary oppositions, as no one was ready to give them space because of their sexual orientation to finally finding a safe space. The tone used by the actors are confident and unapologetic which contrasts with the historical hiding of queer love to public declaration. The hashtag #FreeToLove functions as a semiotic cue that outdo traditional gender boundaries (Gusfa and Ramdani, 2021). A shift from the historical tendency to conceal queer relationship to open and public affirmation of commitment is illustrated in the advertisement. The advertisement is set in urban context, specifically focusing on house hunting. The couple face a lot of rejection and hatred but eventually finds a place where they were accepted and treated just like any other heterosexual couple. The choice of setting and depiction of queer couple significantly contribute to the transmission of intended message in visual discourse. The employment of direct eye contact operates as a potent device facilitating an interpersonal interaction that captures the viewer's attention and encourages engagement with the communicated message.

#### 5.3.2 Signified associations

The lack of safe space for the queer bodies in India metaphorically represents to social exclusion. “After so many rejections, we finally found a landlady who accepted us” suggests the eventual acceptance, signifying normalization and recontextualization of domestic space as a site of belonging rather than rejection. The landlady's acceptance functions as a metaphor for allyship, recontextualizing domestic space as a site of belonging rather than exclusion. The campaign is presented in non-fiction or biographical narrative featuring queer individuals sharing authentic experience but there is no evidence regarding the identity of the actors. The hashtag #FreeToLove reconceptualizes love as a socially valuable construct beyond gender stereotypes. This strategy reflects what Sender (2004) identifies as the “mainstreaming” of queer identity, making queerness legible and acceptable to heterosexual consumers by emphasizing sameness over difference.

### 5.4 Infosys “Embrace with Love”- 2022

#### 5.4.1 Identifying semiotic resources

Infosys (2022) Pride campaign uses animation to negotiate queer visibility in a corporate framework. Storytelling style is used, focusing on the emotional journey of fitting within a heteronormative society. The linguistic resource includes the caption of the advertisement, which says “Don't let the world tell you who you are. Or should be. Choose any color, identity you feel comfortable with. We're all different. We're all similar. Let's embrace it with Pride,” encourages people to accept themselves first. Unlike other ads which uses influencers or real people for authenticity, Infosys chose an animated story centered on a queer character. The animation functions as a semiotic resource for abstraction. To simplify social tensions the creator uses animation to find a community without the constraints of realistic settings. The protagonist is a male who is depicted as an individual forced to conform to stereotypical gender norms. He is denied anything considered “feminine.” A bow replaced with a cap, a doll with a miniature soldier, and ballet dismissed as inappropriate. As he grows up, the narrative links these childhood preferences to his being stopped from falling in love with another man. The advertisement critically conflates two analytically distinct categories: gender expression and sexual orientation. By coding the protagonist's love of ballet and feminine-coded objects as markers of his gay identity, the campaign inadvertently reinforces the harmful stereotype that gay men are inherently effeminate—what Butler (1990) identifies as the “heterosexual matrix,” in which gender presentation is assumed to map onto sexual orientation. This conflation equally risks equating the experience of transgender individuals with that of cisgender gay men, erasing the distinct material, social, and medical realities each community navigates (Serano, 2007). This representational slippage may indicate limitations in the campaign's engagement with the diversity of LGBTQ+ experiences. He navigates isolation until he finds an accepting community. The protagonist has a rainbow heart which is repeatedly changed by a hand into “blue,” the color set as a symbol for males. The hand represents the society which suppresses the true colors. The background song is also an important semiotic element as the lyrics goes “I'm every color, every color that you know… every color of the rainbow, don't let go,” signifying the self-acceptance. This campaign being animated has the risk of remaining in the realm of the sweet story rather than addressing material legal struggles.

#### 5.4.2 Signified associations

The storyline of the protagonist who finally finds a community signifies the recontextualization of the workplace. The signified association of a global IT corporation is shifted from a cite of rigid hierarchy to a safe space of belonging to the queer community. The “rainbow heart” signifies the identity of the protagonist and the blue heart signifies the societal stigma placed upon him. The stereotypical belief of blue being associated with males is the reason behind the usage of blue heart. The hand that keeps on stopping the protagonist from choosing what he likes denotes the biased society. At one point the hand could not replace the beautiful rainbow heart and that is when the protagonist finally breaks away from social constrains and chooses his own path. The choice of animation may signify a refusal of the visual validation of photography. The campaign presents a polished version of inclusion that centers on acceptance rather than subjugation.

### 5.5 Amazon Prime “Happy Pride Month”-2023

#### 5.5.1 Identifying semiotic resources

The MCDA of Prime Video India's (2023) “Happy Pride Month” campaign reveals how the brand uses archival media fragments from different movies to negotiate queer visibility and corporate allyship in contemporary India. Queer moments from various movies like *Made in Heaven, Uncle Frank, Shubh Mangal Zyada Saavdhan, Modern Love, The Boys, Guilty Minds, Happy Family Conditions Apply, Maja Ma*, and *Four More Shots Please* were assembled together and recontextualized by moving the narrative from a fictional context to a strategic discourse of social legitimization. The fragments bought in as a whole exemplify systematic intertextuality, where preestablished characters become semiotic indicators of the brand's evolving socio-political identity. This advertisement uses confessional style phrases like “I was in school… when I first realized that I was gay” and “I'm a Lesbian! I don't like men, I like women!” to show the shift from hiding to declaration. The fragment from *Maja Ma*, “Let Live! Let us have a life too!” functions as a rhetorical intervention demanding fundamental human rights. The tone of the fragments used in the advertisement move from fear of expression as shown in *Made in Heaven* as “I was so scared, I couldn't admit it” to triumphant affirmation of lesbian identity in Four *More Shots Please*. This tonal shift provides emotional coherence to the message of pride.

#### 5.5.2 Signifies associations

The metaphorical journey from being a taboo subject to being socially visible is signified in the final fragment of the advertisement, where the actress playing a celebrity role in Four *More Shots Please* publicly accepts her identity as a lesbian. The recontextualization of Indian family script is observed in the fragment from *Happy Family Conditions Applied* “I hand over my Sanu… to you, son,” where the father entrust his son to his lover. In India, families are often seen as the judge of queer relationships but in this case, it shifts from a potential site of rejection to a space where queer identity is normalized and accepted. Prime Video positions itself as a cultural intermediary that seeks to align its corporate identity with discourses of LGBTQ+ inclusion

### 5.6 Ideology and power relations

Across all five advertisements, a shared neoliberal ideology operates through which brands gain social legitimacy by aligning commercial interests with progressive causes, while the structural conditions of LGBTQ+ communities remain largely unaddressed. While most transgender ads in India focus on human rights and struggle, ads featuring same-sex relationships predominantly frame the issue as a journey toward emotional acceptance rather than a legal battle (Jetubhai, 2024). Borosil challenges cis-heteronormativity by “denaturalizing” the assumption that Valentine's Day is exclusive to straight couples (Machin and Mayr, 2025), yet simultaneously reflects a neoliberal discourse where identity and liberation are tied to consumption. OkCupid similarly occupies a moral high ground by using inclusive pronouns and positioning the platform as an ally against bias. However, this framing reinforces homonormativity (Duggan, 2002), reducing queer liberation to mainstream-palatable goals such as “universal love,” while sidelining structural struggles unaddressed in corporate Pride discourse. These sidelined struggles include, concretely, anti-discrimination legislation in employment and housing, access to transgender healthcare, and legal recognition of chosen family structures, none of which feature in any of the five advertisements studied here. Closeup denaturalizes the notion that only heterosexual relationships are valid, yet by depicting fluent, English-speaking characters in metropolitan settings, the campaign reinforces an urban and affluent bias. Infosys goes further by critically conflating gender expression with sexual orientation, coding the protagonist's love of ballet and “feminine” objects as markers of his gay identity, inadvertently reinforcing the “heterosexual matrix” (Butler, 1990) and risking the erasure of the distinct material and social realities navigated by transgender individuals (Serano, 2007). The animation's rainbow-washing substitutes colorful symbolism for substantive engagement with queer lived experience, consolidating a power dynamic where visibility remains a feature of the elite, technology-driven urban class. Amazon Prime's campaign similarly leans into what Gould (2009) terms “strategic visibility,” deploying queer imagery during Pride Month as reputational capital while remaining disengaged from year-round political advocacy. Across all five ads, the surge in LGBTQ+ representation mirrors the broader shift in power relations following the decriminalization of Section 377 in 2018, yet visibility without corresponding structural change risks functioning primarily as an aesthetic expression of rainbow capitalism rather than substantive political engagement. These findings suggest that contemporary queer advertising in India functions simultaneously as a site of inclusion and a mechanism of market incorporation.

## 6 Discussion

The following section brings together findings from both the quantitative survey and the MCDA analyses to address the overarching research question. Rather than restating individual advertisement findings, the discussion synthesizes patterns across both methodological strands to draw broader inferences about negotiated queer visibility in Indian commercial media.

### 6.1 Neoliberal ideology and the paradox of rainbow capitalism

The presence of neoliberal ideology is one of the recurring themes in the MCDA of queer advertisements. In this, identity and freedom are intrinsically connected to consumption. The result of quantitative factor analysis complements the qualitative study as a strong construct related to skepticism toward corporate involvement is observed. The survey findings suggest that audiences are highly aware of the commercial motives and views Pride marketing as exploitative. The inference drawn is, while the visibility is valued by the audience, they remain cautious of personal struggles being commodified into consumables.

Campaigns like Amazon Prime and Infosys noted for their performative activism. They often focus on queer liberation only during Pride Month without year-round commitment to legal struggles. The survey's Factor 1 of Table 2 reveals deep skepticism toward corporate motives. High factor loading for profit-driven branding (0.845) and exploitative marketing (0.765) indicate the audience are aware of commercial intend of the brand. Pearson Correlation analysis shows that increased media consumption correlates with both positive perception and heightened skepticism. This reveals that the more the audience engage with these narratives, the more they appreciate visibility but at the same time they do critique the market intent.

### 6.2 Normalization

A transformation in the narrative from concealment to public celebration is explained through qualitative analysis. This shift signifies a critical advancement in social integration, where queer identities can be openly expressed and recognized. Brands such as Borosil and OkCupid use metaphors of “firsts” such as #FirstValentine and #LoveAtFirstPride to signify the social rebirth of queer community following the decriminalization of Section 377. The qualitative shift toward biographical legitimization using real experiences is validated by data in Factor 2, Representation improved (0.757) in Table 2, illustrating a coherent construct of normalization and acceptance. The qualitative analysis shows how brands utilize sophisticated semiotic resources to normalize queer identities and the quantitative data highlights a critically engaged audience that acknowledges progress while remaining skeptical of corporate motives.

### 6.3 Recontextualizing the Indian family setup

The qualitative analysis of Closeup and Amazon Prime advertisements highlights a significant move toward recontextualizing domestic space. By placing the queer identities in an inclusive family setting denaturalizes heteronormative dominance in the traditional Indian family structure. The socially constructive element helps destigmatize identities for a mainstream audience, even though the primary goal remains profit-oriented for the corporation.

### 6.4 Representational limits and urban elite bias

As established in the MCDA analysis (Section 5.6), all five advertisements situate queer visibility within affluent, metropolitan, English-speaking settings. The quantitative data now adds an audience dimension to this finding, that is, the strong positive correlation between “Limited Identities Focus” and “Profit-driven Branding” (Table 4) suggests audiences read this narrowness not as oversight but as deliberate targeting. Taken together, the quantitative and qualitative findings converge on a central tension: corporate queer advertising in post-2018 India produces genuine cultural visibility while simultaneously containing it within neoliberal, urban, and consumption-oriented frameworks. The audience is neither passive nor uncritical as survey data consistently shows that appreciation for visibility coexists with sharp skepticism toward commercial motives. This negotiated reception mirrors the negotiated visibility the advertisements themselves perform: inclusion and commodification not as opposites, but as co-constitutive features of rainbow capitalism in the Indian context.

## 7 Conclusion

The transition of queer identities in Indian media from being criminalized to one of prominent commercial visibility marks a landmark shift that is both empowering and exploitative. The decriminalization of Section 377 in 2018 serves as the catalyst for a landmark transformation in media representation. This research article has used a mixed-method approach combining MCDA with quantitative audience survey to draw inferences on negotiated visibility where inclusion and Pride marketing coexist. The qualitative findings reveal that the brands use semiotic normalization to embedded queer identities within familiar cultural narratives, recontextualizing the Indian family script and making the audience accept is as a normal part of everyday life. The findings suggest that queer-themed advertising functions as a double-edged phenomenon. They simultaneously facilitate social visibility and incorporate queer identities into market-oriented frameworks.

The quantitative insists that the audience are neither passive or uncritical. The acceptable reliability of Cronbach α = 0.780 and significant positive correlations, the data confirms that the general public does recognize and appreciate increased visibility of queer identities. A substantial proportion of the respondent's express skepticism toward corporative motives and the commodification of queer narratives, highlighting concerns about rainbow capitalism and dilution of activist intent. These findings underscore a central paradox of queer visibility in contemporary media being both empowering and circumscribed. It contributes to social normalization and border acceptance and mediates neoliberal logics that prioritize marketability over inclusivity. This study therefore argues that queer visibility in commercial advertising should be evaluated not only by its presence, but also by the political, social, and economic conditions under which that visibility is produced.

## Data Availability

The original contributions presented in the study are included in the article/supplementary material, further inquiries can be directed to the corresponding author.

## Appendix

**Queer Representation Media and Literature**

1. Age
  - 17–19
  - 20–25
  - 26–30
  - Above 30
2. Sex
  - Prefer not to say
  - Male
  - Female
3. Highest qualification
  - Doctoral degree
  - Master's degree
  - Professional degree
  - Bachelor's degree
  - School education
  - Others
4. How often do you consume media (TV shows, movies, books) that feature queer themes?
  - Always
  - Often
  - Sometimes
  - Rarely
  - Never
5. Queer representation in modern media has improved in recent years.
  - Strongly agree
  - Agree
  - Neutral
  - Disagree
  - Strongly disagree
6. Corporate Pride campaigns (e.g., rainbow branding, sponsorships) are genuine efforts to support LGBTQ+ rights rather than just a marketing strategy.
  - Strongly agree
  - Agree
  - Neutral
  - Disagree
  - Strongly Disagree
7. It's good that corporations are launching Pride-themed products.
  - Strongly agree
  - Agree
  - Neutral
  - Disagree
  - Strongly disagree
8. Queer characters in media are written in a way that appeals to straight audiences rather than representing authentic experience.
  - Strongly agree
  - Agree
  - Neutral
  - Disagree
  - Strongly disagree
9. Queer stories in commercial media tend to focus on a few identities (e.g., white gay men) while ignoring others (e.g., trans, nonbinary, queer people of color).
  - Strongly agree
  - Agree
  - Neutral
  - Disagree
  - Strongly disagree
10. While commercialization has increased global visibility for queer narratives, it has also diluted their original meaning and activism.
  - Strongly agree
  - Agree
  - Neutral
  - Disagree
  - Strongly disagree
11. Corporate branding and Pride-themed merchandise are more about profit than actual support for LGBTQ+ rights.
  - Strongly agree
  - Agree
  - Neutral
  - Disagree
  - Strongly disagree
12. The rise of LGBTQ+ branding and marketing during Pride Month is more exploitative thansupportive.
  - Strongly agree
  - Agree
  - Neutral
  - Disagree
  - Strongly disagree
